# DWI-MRI: Single, Informative, and Noninvasive Technique for Prostate Cancer Diagnosis

**DOI:** 10.1100/2012/973450

**Published:** 2012-02-14

**Authors:** Elhousseiny I. Ibrahiem, Tarek Mohsen, Adel M. Nabeeh, Yasser Osman, Ihab A. Hekal, Mohamed Abou El-Ghar

**Affiliations:** ^1^Urology Department, Urology and Nephrology Center, Mansoura University, Mansoura 35516, Egypt; ^2^Radiology Department, Urology and Nephrology Center, Mansoura University, Mansoura 35516, Egypt

## Abstract

*Aim*. To evaluate diffusion weighted image-MRI (DWI) as a single diagnostic noninvasive MRI technique for prostate cancer (PCa) diagnosis. *Material and Methods*. A prospective study was conducted between July 2008 and July 2009. Candidates patients were equal or more than 40 years old, with suspicious digital rectal examination (more than clinical T2) or PSA >4 ng/mL. Informed consent was signed. DWI-MRI was performed at 1.5 T with a body coil combined with a spine coil in consecutive 100 cases. The histopathology of biopsies has been used as reference standard. Two examiners were evaluating MRI and TRUS, both of them were blinded regarding pathological findings. Accuracy, specificity, and sensitivity were statistically analyzed. *Results*. Based on pathological diagnosis: group A (cancerous); 75 cases and group B (non-cancerous); 25 cases. Mean age was 65.3 and 62.8 years in groups A and B, respectively. Mean PSA was 30.7 and 9.2 ng/mL in groups A and B, respectively. Sensitivity of DWI was 58.3% while specificity was 83.8%. Accuracy of lesion detection was 52.4–77.8% (*P* < 0.05). Moreover, DWI at ADC value 1.2 × 10^−3^ mL/sec could determine 82.4% of true positive cases (*P* < 0.05). ADC values were lower with Gleason score ≥7 (*P* < 0.05). *Conclusion*. DWI could represent a non invasive single diagnostic tool not only in detection and localization but also in prediction of Gleason score whenever DWI is used prior to invasive TRUS biopsy. Furthermore, targeted single biopsy could be planned after DWI to minimize patient morbidity by invasive techniques.

## 1. Introduction

As it is known, prostate cancer is a major health problem, and exploration of noninvasive imaging methods that have a high specificity and sensitivity is still critically needed. 

Magnetic resonance imaging (MRI) has been widely used for pretreatment workup in patients with prostate cancer (PCa). Newly applied MRI techniques as diffusion weighted image (DWI) are considered as a promising technology. DWI provides functional information about the behavior of water molecule in the tissue. DWI offers more power to evaluate PCa through various points: detection [1], localization [2], and tumor aggressiveness [3].

In order to assess this value simultaneously in one group of patients, a prospective study was conducted. Our aim is to study the value of DWI as single technique in high-risk patients (elevated PSA and more than clinically T2) as fully informative diagnostic tool.

## 2. Material and Methods

### 2.1. Patients

A prospective ethically approved study was conducted on 100 patients. Inclusion criteria were patients more than or equal to 40 years, aged abnormal digital rectal examination (more than clinical T2), PSA ≥4 ng/mL with signed consent. Patients with prior PSA measurements and biopsy, prostatic operations, or positive family histories of PCa with previous transrectal ultrasound (TRUS) were excluded. This study was carried out between July 2008 and July 2009. Diagnosis was confirmed by histopathology through TRUS biopsy. The interval time between MRI (earlier) and TRUS biopsy (later) ranged between14 and 21 days (mean: 16.7 days).

### 2.2. MR Technique

Patients underwent MRI at 1.5-T MRI (SIGNA Horizon, General Electric Medical Systems, Milwaukee, WIS) using surface coil. Initially, high spatial resolution T2W images of the prostate were obtained using TR = 7.000–8.000 ms, TE = 90–102 ms, band width = 20–83 kHz, 256 × 256 matrix, slice thickness of 3 mm, intersection gap of 1 mm, and field of view (FOV) = 20 cm. DWI were then obtained, under free breathing, using monodirectional gradients and a multisection fast spin-echo-type (FSE) echoplanar sequence in the axial plane by using a body coil with the following parameters: TR = 8.000 ms, TE = 61.2 ms, band width = 142 kHz, 256 × 256 matrix, slice thickness of 5 mm, intersection gap of 0 mm, and FOV = 36 cm, seven excitations, water excitation with *b* value of 0 and 800/mm2. Thirty to 54 slices were obtained in 60–120 sec. to cover the pelvis in each patient.

### 2.3. Image Analysis

Image was analyzed using the function tool software (General Electric Medical System, Milwaukee, Wisconsin). Blinded to the results of clinical and histopathology data, two radiologists interpreted the MRI images. Discrepancies and equivocal cases were resolved by consensus either positive or negative. PCa appeared on DWI with a *b* value of 800/mm^2^ as high signal intensity relative to bladder wall and the surrounding urine. Any lesion on peripheral zone has low apparent diffusion coefficient (ADC) value than 1 × 10^−3^ mm^2^/sec. is considered negative. In our study, for statistical purposes, focal lesion was identified by tumor involved one lobe with <3 cores and multifocal lesion was bilateral distribution of the positive cores or involvement of more than 3 cores.

### 2.4. Diagnostic Criteria

When DWI was used, any prostatic lesion showing a decrease on ADC map with fluid restriction (high SI at *b* = 800 and low SI at *b* = 0) was considered malignant (Figure 3). The regions of interest circles were placed in the suspected areas as large as possible for cancerous zones and at random more than twice in each benign zone, and the mean value was calculated as the value of ADC for each noncancerous zone. ADC value of each zone was recorded, and the series was arranged in order. The prostate was divided, corresponding to biopsy sites into 6 regions on MRI image to facilitate the localization accuracy comparison.

### 2.5. Prostate Biopsy

All patients underwent TRUS guided biopsy (12-cores: apical, midzonal and, basal; 2 biopsies per each zone). Whenever PSA was high and biopsies were negative, rebiopsy was done after 2 weeks. The TRUS machine: B and K medical panther (7.5 MHz Bruel and Kjaer, model 2002 (Naerum, Denmark)). A local anesthetic (2% lidocaine jelly (10 mL)) has been instilled intrarectally ten minutes before TRUS. Biopsy was taken with 18 G needles driven by a spring-loaded gun. An individual histological analysis for each sample site was performed. The PCa locations were assigned definitely corresponding zones.

### 2.6. Statistical Analysis

All data were analyzed using SPSS 16.0 for Windows (SPSS Inc., Chicago, IL, USA). ADC value was evaluated by means of receiver operator characteristics (ROC) analysis. The area under the ROC curve was calculated for comparison. Sensitivity, specificity, positive predictive value (PPV), and negative predictive value (NPV) for DWI had been calculated in order to evaluate the reliability. Chi-square test was used when appropriate, with consideration of statistical significance at *P* < 0.05.

## 3. Results

On histopathological examination of 1692 prostatic biopsies (100 cases): 75 cases were harboring one or more cancerous focus in their biopsies (group A) and 25 cases proved to be noncancerous (group B). Within group B, 15 cases had BPH and 10 cases had chronic prostatitis. Forty-one cases had been subjected to second set biopsies.

Only 92 cases completed the DWI-MRI study (68 cases in group A, and 24 cases in group B). Mean age of the studied group was 65.03 years (SD ± 7.13 years). Mean PSA was 26.3 ng/mL (SD ± 24.2 ng/mL). Mean volume of the gland was 60.09 grams (SD ± 28.7 grams). According to radiological TNM staging, 13/68 cases were T3 prostate cancer. While 16/68 cases were stage T4.

DWI-MRI was able to identify 67/92 cases (72.8%) as positive cases. However, 25/92 cases (27.2%) was identified as negative of malignancy. Diagnosis of DWI was in agreement with malignant histopathological finding in 57 cases while 14 cases were negative in correlation with their corresponding nonmalignant histopathology. The specificity was 83.3%, sensitivity was 58.3%, PPV was 85.1%, NPV was 56%, and accuracy was 77.2%.

Localization of the tumor focal lesion was in agreement with histopathology map in 11/21 cases (52.4%); however, the remaining 10/21 cases were seen as multifocal lesions. While 28/36 cases (77.8%) were identified as multifocal when they were compared with the histopathological map, *P* < 0.020.

**Figure 1:**
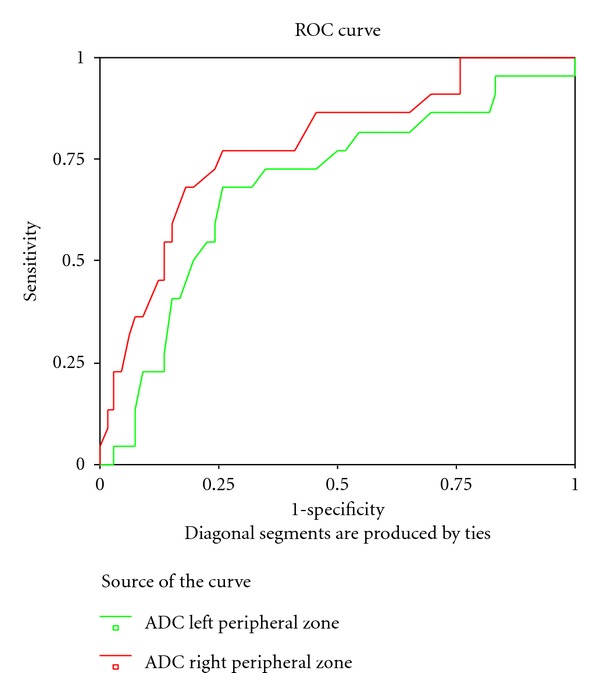
Receiver operating characteristics (ROC) curve of ADC values of peripheral prostatic zones (ADC of right peripheral zone: area under the curve: 0.781, *P* = 0.0001, 95% confidence interval: 0.666–0.895; ADC of left peripheral zone: area under the curve: 0.682, *P* = 0.011, 95% confidence interval: 0.547–0.816).

**Figure 2:**
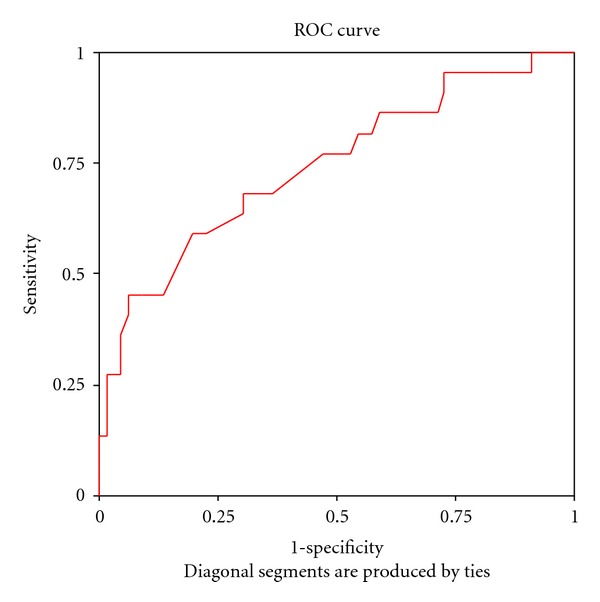
Receiver operating characteristics (ROC) curve of ADC values of central prostatic zones (ADC of central zone: area under the curve: 0.749, *P* = 0.001, 95% confidence interval: 0.623–0.874).

**Figure 3:**
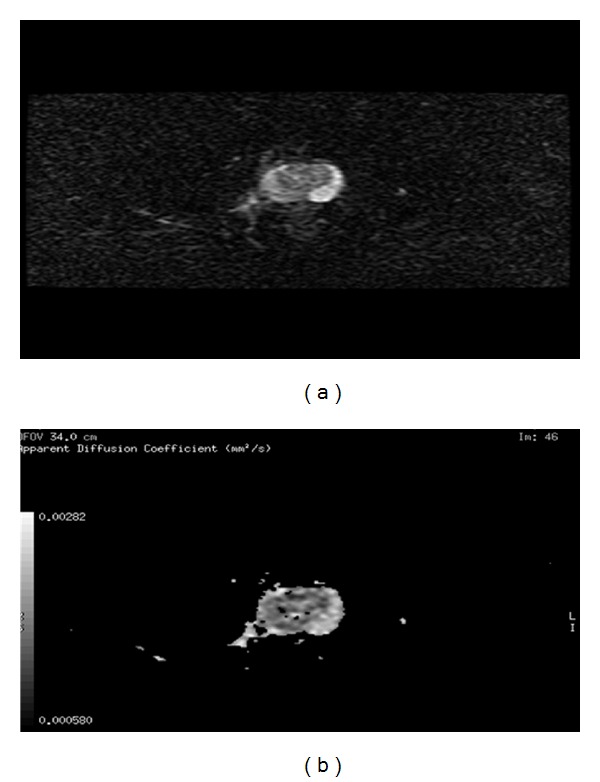
Example of 62-years-old male patient with cancer prostate and PSA >10 ng/mL. At the corresponding DWI/ADC map (a) there is a relative hyperintense lesion at the medial aspect of the right PZ at *b* = 800 and it is hypointense at *b* = 0 and at ADC map (b).

Receiver operating characteristics (ROC) curve of ADC values of different prostatic zones was performed to define the most reliable cutoff point to differentiate cancerous and noncancerous conditions. ADC value of right peripheral zone: area under the curve: 0.781, *P* = 0.0001, 95% confidence interval: 0.666–0.895, ADC of left peripheral zone: area under the curve: 0.682, *P* = 0.011, 95%  confidence interval: 0.547–0.816 Figure 1. ADC value of central zone: area under the curve: 0.749, *P* = 0.001, 95% confidence interval: 0.623–0.874, Figure 2. 

To differentiate cancerous and noncancerous status: at commony used ADC cutoff value 1 × 10^−3^ mm^2^/seconds, the sensitivity was ranged between 81.8 and 86.4% and 86.4% on peripheral (right and left zones) and central zone, respectively. The specificity was 41.5–43.1% on peripheral zone and 29.2% on central zone. However, at ADC cutoff value 1.2 × 10^−3^ mm^2^/sec.; sensitivity ranged between 72.2 and 86.4%, with specificity range between 50.8–63.2%. While the central zone reached 86.4% sensitivity and 38.5% specificity at the same cutoff point. Furthermore at ADC value 1.5 × 10^−3^ mm^2^/sec., the sensitivity was 68.2–77.3% and specificity range was 73.8–75.4% on peripheral zones. On the prostatic central zone at the same value, the sensitivity was 68.2% and specificity was 70.2% (*P* < 0.05).

Of clinical importance, the ADC value was correlated with the pathological Gleason score.  Table 1 shows a statistical significant difference between Gleason score less than 7 and ADC value around 1.4 × 10^−3^ mm^2^/sec. in all measured zones, *P* < 0.05. In other words, ADC value less than 1.4 × 10^−3 ^mm^2^/sec. was associated with Gleason score more than or equal to 7, *P* < 0.05.

## 4. Discussion

MRI is the imaging tool of choice in the evaluation of prostate cancer. DWI-MRI has many advantages over other MRI techniques, it is non invasive (unlike contrast enhanced or endorectal MRI), acquisition time is less than 2 minutes (unlike spectroscopy), and has good specificity (unlike T2-Weighted MRI). However, the limitations are its poor spatial resolution and the potential risk of image distortion because of hemorrhages after prostatic biopsy [4].

In high-risk patients (elevated PSA and clinically more than T2) who have a limited surgical chance. Especially with late tumor stages, precise delineation of tumor is essential for radiotherapy planning [2]. This will be enhanced in high-risk patients as MRI detection of PCa is dependent on tumor size [5, 6].

Furthermore in order to facilitate targeted biopsy which has a higher detection rate than conventional biopsies [7], avoiding unnecessary biopsies without missing PCa in men with elevated PSA rather than avoiding annoying biopsies-related complications are aspects that require focus.

In our study, DWI has the ability to identify tumor in comparison to malignant histopathology. Detection ability has specificity 83.8%, sensitivity 58.3%, PPV 85.1%, NPV 56%, and accuracy 77.2%. Our results were in agreement with Shimizu et al. [8] (DWI has sensitivity 56.7% and PPV was 86.4%). While Chen et al. [9] reported high sensitivity and specificity (82.4%, 81.6%, resp.).

In our study we found accuracy of tumor detection on DWI-MRI was high (82.4%, *P* < 0.05) with ADC values around 1.2 × 10^−3^ mm^2^/sec. Chen et al., in their study, found that ADC value less than 1.3 × 10^−3^ mm^2^/sec. increased specificity and sensitivity of tumor detection to 81.6 and 82.4% [9]. No studies in English literature discuss localization power of DWI as sole noninvasive MRI. Localization sensitivity and specificity were 84%, 82%, DWI endorectal coil in study of Yağci et al. [10]. In our result, localization of the cancerous focal lesion was in agreement of histopathology map in 52.4% while multifocal were identified in 77.8% accurately in our study. However, based on our result, we assume that DWI has more precise localization whenever more than 3 foci are presented. According to our results we suppose that whenever DWI is used prior to invasive biopsy, and through its role towards targeted biopsy (localization accuracy), it could save time and cost and lower the complications that may be yielded by biopsy.

Woodfield et al. suggested an inverse relationship between ADC value of DWI and Gleason score [11]. We agreed with them and also we concluded ADC value 1.4 × 10^−3^ mm^2^/seconds as cutoff point that can discriminate between low-risk (Gleason ≤ 6) and high-risk (Gleason ≥ 7) prostate cancer.

Among our cases, 56% of them had high Gleason score and all cores are positive in 62.7% of cases. Shimizu et al. documented that sensitivity of tumor detection by MRI is increasing with tumor size and Gleason score [8]. However, they found that PPV of DWI and T2W was 86.4% and 83%. While in our study PPV were 85.1% and 81.1% for DWI and T2W, respectively.

In brief, DWI as single noninvasive tool is an informative MRI modality. It was able to answer questions of localization, tumor detection, and predication of pathological Gleason scores. Targeted single biopsy could be planned after DWI to minimize patient morbidity by invasive techniques.

A limitation of our study is that we compared DWI with histopathology biopsy results and not with prostatectomy specimens. This was due to the limited cases have been operated on. In view of high-risk sector of our studied patients (elevated PSA, more than clinical T2) with high radiological staging (T3 or more: 42.6%), all these factors hinder the possibility of radical prostatetcomy.

## 5. Conclusion

DWI could represent a non invasive single diagnostic tool not only in detection and localization but also in prediction of Gleason score. DWI has acceptable identification power (accuracy was 77.2% and was specificity 83.8%). DWI could localize multifocal lesion (77.8%) better than focal lesion (52.4%). Detection rate was better with ADC value 1.2 × 10^−3^ mm^2^/seconds, as cutoff value, than 1 × 10^−3^ mm^2^/seconds (specificity: 50.8–63.2%). Lastly, on ADC value: 1.4 × 10^−3^ mm^2^/seconds, DWI can differentiate Gleason score more or less than 7.

##  Conflict of Interest 

The authors declare that there is no conflict of interests.

## Figures and Tables

**Table 1:** Correlation between ADC values and Gleason score.

	ADC right peripheral zone mean (± SD)	ADC left peripheral zone mean (± SD)	ADC central zone mean (± SD)
Gleason score ≤6 (*n* = 28)	1.49 (± 0.437)	1.4656 (±0.509)	1.415 (± 0.382)
Gleason score ≥7 (*n* = 39)	1.045 (± 0.366)	1.003 (± 0.387)	1.219 (± 0.426)
*P* value	0.0001	0.0001	0.060
